# The Correlation between Optimism and Multidimensional Sleep Health in Adults Aged 65 and Older

**DOI:** 10.1093/geroni/igaf122.3104

**Published:** 2025-12-31

**Authors:** Brooke Sullivan, Caitlan Tighe

**Affiliations:** Providence College, Providence, Rhode Island, United States; Providence College, Providence, Rhode Island, United States

## Abstract

Optimism has been explored in conjunction with sleep and sleep-related experiences in adults. Fewer studies have examined these associations among older adults specifically and most have investigated associations with single dimensions of sleep or insomnia symptoms. The current study adds to the literature by investigating associations of optimism, positive and negative emotions, and multidimensional sleep health in adults aged 65 years and older. We investigated the study aims with data from a cross-sectional online survey study. The sample included 179 participants ranging in age from 65 to 82 years old (M = 69.7, SD = 4.2). The Comprehensive Inventory of Thriving was used to measure optimism, positive emotions, and negative emotions. The RU_SATED V4.0 Questionnaire was used to measure multidimensional sleep health. Pearson correlations were used to test bivariate associations between multidimensional sleep health and optimism, positive emotions, and negative emotions, respectively. Higher levels of optimism were significantly associated with better multidimensional sleep health (r =.31, p<.001). Higher levels of positive emotions (r =.34, p = <.001) and lower levels of negative emotions (r =-.32, p<.001) were associated with better multidimensional sleep health. Taken together, these findings point to links between optimism, emotional experiences, and self-reported multidimensional sleep health among older adults. Future studies may investigate possible causal links between optimism, emotional experiences, and multidimensional sleep health and, ultimately, attempt to determine if interventions to improve optimism and positive emotional experiences could also improve multidimensional sleep health among older adults.

